# Tolerogenic Dendritic Cells Reduce Cardiac Inflammation and Fibrosis in Chronic Chagas Disease

**DOI:** 10.3389/fimmu.2020.00488

**Published:** 2020-04-07

**Authors:** Emanuelle de Souza Santos, Luciana Souza de Aragão-França, Cássio Santana Meira, Jéssica Vieira Cerqueira, Juliana Fraga Vasconcelos, Carolina Kymie Vasques Nonaka, Lain Carlos Pontes-de-Carvalho, Milena Botelho Pereira Soares

**Affiliations:** ^1^Gonçalo Moniz Institute, FIOCRUZ, Salvador, Brazil; ^2^Center for Biotechnology and Cell Therapy, Hospital São Rafael, Salvador, Brazil

**Keywords:** antigen presenting cells, Chagas disease, inflammation, fibrosis, *Trypanosoma cruzi*, cardiomyopathy

## Abstract

Chronic Chagas disease cardiomyopathy (CCC) is the most frequent and severe form of this parasitic disease. CCC is caused by a progressive inflammation in the heart, resulting in alterations that can culminate in heart failure and death. The use of dendritic cells (DCs) appears as an option for the development of treatments due to their important role in regulating immune responses. Here, we investigated whether tolerogenic cells (tDCs) could interfere with the progression of CCC in an experimental model of Chagas disease. The tDCs were generated and characterized as CD11b^+^ CD11c^+^ cells, low expression of MHC-II, CD86, CD80, and CD40, and increased expression of PD-L. These cells produced low levels of IL-6 and IL-12p70 and higher levels of IL-10, compared to mature DCs (mDCs). Interestingly, tDCs inhibited lymphoproliferation and markedly increased the population of FoxP3^+^ Treg cells *in vitro*, compared to mature DCs. In a mouse model of CCC, treatment with tDCs reduced heart inflammation and fibrosis. Furthermore, tDCs treatment reduced the gene expression of pro-inflammatory cytokines (*Ifng* and *Il12*) and of genes related to cardiac remodeling (*Col1a2* and *Lgals3*), while increasing the gene expression of IL-10. Finally, administration of tDCs, increased the percentage of Treg cells in the hearts and spleens of chagasic mice. Ours results show that tolerogenic dendritic cells have therapeutic potential on CCC, inhibiting disease progression.

## Introduction

Chagas disease is caused by the protozoan parasite *Trypanosoma cruzi*, and affects about 8 million people worldwide, mainly low income populations (1). Although considered a neglected tropical disease endemic in Latin America, this disease is spreading to other continents, such as Europe, North America, Asia and Oceania, due to human migration (2, 3). The acute phase of Chagas disease is characterized by high parasitemia with immune-inflammatory response to the parasite, which result in organ damage (4). After the initial infection, most of the patients remain asymptomatic without clinical manifestations. About 20–40% of the chronic patients, however, develop the cardiac and/or digestive forms of the disease, for which there are no available treatments (5, 6).

The chronic Chagas disease cardiomyopathy (CCC) is most frequent and severe manifestation found, with symptoms that range from mild to severe cardiac remodeling associated with inflammation, fibrosis, arrythmias and thromboembolic events, which may lead to congestive heart failure and sudden death (7, 8). In addition, microvascular ischemia is directly involved in the histopathological pattern of CCC, which consists of a diffuse focal myocarditis associated with myocytolysis and fibrosis (5).

The only available treatment for end-stage CCC patients is heart transplantation, a high-cost procedure not available in time for all patients. Furthermore, organ transplantation presents several complications in patients with chronic infection due to immunosuppressive therapy, which may trigger reactivation of infection (4, 7, 9). Alternative treatments are needed, with the aim of reducing morbidity and mortality, and providing a better quality of life for the patients with CCC. Therapeutic alternatives focused on reducing the immunological mechanisms, which are key factors in the pathogenesis of the disease, modulating both inflammation and fibrosis in the heart of the patients, are of great interest (10–16).

In this context, immunotherapy with dendritic cells (DCs) appears as an option for the development of treatments based on their role in immune responses. Dendritic cells are APCs which can activate T cells and induce T cell tolerance (15). They are important as mediators for the induction of effective immunity against invading pathogens, by connection of innate and adaptive immune responses and maintenance of immune tolerance (17, 18). Dendritic cells (DCs) represent a heterogeneous population of potent APCs that include multiple subsets which different functional specializations that depends to their origin, maturation state, location, and environmental conditions (18, 19). The role of DCs in induction of immunity or tolerance is determined by their maturation state (17). Mature DCs mediate immune responses under inflammatory conditions, whereas immunological tolerance is induced by DCs with regulatory profile, characterized by an immature phenotype (19), with low expression of co-stimulatory and MHC molecules, distinct cytokine profile and expression of inhibitory molecules (18).

Considering the role of DCs in both the induction of adaptative immunity and in maintaining immunological tolerance, much attention has been given to the role of these cells in stimulating or preventing autoimmune diseases, associating the potential application of DCs as regulators for treatments of autoimmune diseases. Thus, tolerogenic dendritic cells (tDCs) are a promising therapeutic tool to reduce or prevent autoimmune diseases, including systemic lupus erythematosus (20), atherosclerosis (21), asthma allergic (22), rheumatoid, and inflammatory arthritis (23), experimental autoimmune myocarditis (24), myocardial infarction (25), immunotherapy of cancer (26), and solid organ transplantation (19).

In the present study, we aimed to investigate whether tolerogenic dendritic cells can modulate the inflammation in a mouse model of CCC. We show here that immunotherapy with tolerogenic dendritic cells are able to ameliorate heart inflammation and fibrosis, two hallmarks of CCC.

## Materials and Methods

### Animals and *Trypanosoma cruzi* Infection

Female C57BL/6 mice were maintained in the animal facility of the Center for Biotechnology and Cell Therapy, Hospital São Rafael (Salvador, Bahia, Brazil), and provided with rodent diet and water *ad libitum*. Trypomastigotes of the myotropic Colombian *T. cruzi* strain were obtained from culture supernatants of infected LLC-MK2 cells. Infection of C57BL/6 mice was performed by intraperitoneal (i.p.) injection of 1000 *T. cruzi* trypomastigotes in saline, and parasitemia was monitored during acute infection. All experiments were carried out in accordance with the recommendations of Ethical Issues Guidelines and were approved by the local ethics committee for animal use under number 017/2017.

### Generation of Dendritic Cells

The protocol used to produce dendritic cells (DCs) was adapted from a previously described methodology (22). Bone marrow from C57BL/6 mice was collected by flushing the femurs with RPMI medium (Sigma-Aldrich). The cells were then cultured in 75 cm^2^ flasks at a concentration of 10^6^ cells/mL in RPMI medium supplemented with 100 mM pyruvate, 200 mM glutamine, 10 mM HEPES, 10% fetal bovine serum (FBS; GIBCO), 50 μg/mL gentamicin, 0.2% NaHCO_3_, and 30% culture supernatant of X-63 cell line (which produces GM-CSF), at 37°C in a 5% CO_2_ atmosphere. To generate tolerogenic dendritic cells (tDCs), dexamethasone (10^−6^ M; Prodome Laboratory, Campinas, Brazil) was added to the medium at the third day of culture. On day 7, tDCs were activated with 1 μg/mL of *Escherichia coli* lipopolysacharide (LPS; Sigma-Aldrich) for 24 h. Control DCs (mDCs) were generated in the same conditions, with the exception of addition of dexamethasone on the cultures.

### Characterization of Dendritic Cells

For immunophenotyping, activated DCs or tolerogenic DCs were incubated with monoclonal antibody (mAb)- fluorochrome or biotin conjugates: anti-CCR7-PerCP, anti-CD11c-FITC, anti-CD11b-PE, anti-CD40-PE, anti-CD80-PE, anti-CD86-PE, anti-MHC-II-biotin, and anti-PD-L1-biotin (eBioscience Inc.; San Jose, CA) or with the corresponding isotype controls for 20 min at 4°C in the dark and washed twice with saline solution with 1% FBS. PE-avidin was added to the cell suspensions previously incubated with biotin-mAb conjugates, for 20 min at 4°C in the dark, followed by washing once with 1% FBS saline solution. For each sample, data from 100,000 cells was acquired by three-color flow cytometry, using a BD LSRFortessa SORP cytometer and a BD FacsDiva v.6.2 software (Becton Dickinson; Heidelberg, Germany). Cell-free supernatants of mDCs or tDCs were collected 24 h after stimulation and stocked at −20°C until used for cytokine measurements. The concentrations of IL-6, IL-10 and IL-12 cytokines were measured by ELISA, using specific antibody kits (R&D Systems, Minneapolis, MN), according to manufacturer's instructions.

### Lymphoproliferation Assay

For lymphoproliferation assay, splenocyte suspensions from infected C57BL/6 mice (3 months post-infection) were prepared in RPMI medium (Sigma-Aldrich) supplemented with 100 mM pyruvate, 200 mM glutamine, 10 mM HEPES, 10% FBS, 50 μg/mL gentamicin and 0.2% NaHCO_3_. Splenocytes (10^5^ cells/mL) were plated in 96 well plates, in quadruplicate, in a final volume of 200 μL, in presence of 2 μg/mL concanavalin A (Con A; Sigma-Aldrich). The mDCs or tDCs were added at 1:10 ratio. After 48 h, cultures were pulsed with 1 μCi of methyl-3H-thymidine (Perkin Elmer, Waltham, MA) and incubated for additional 18 h. The cells were then harvested and the ^3^H-thymidine uptake was determined using a β-plate counter (Multilabel Reader, Finland). An aliquot of cell-free supernatants was collected 24 h after incubation of splenocytes plus mDCs or tDCs for cytokine measurement. Concentrations of IL-2 and IFNγ cytokines were measured by ELISA, using specific antibody kits (R&D Systems), according to manufacturer's instructions.

### CFSE Staining

Splenocytes from infected C57BL/6 (3 months post-infection) mice were plated into 24-well plates at a cell density of 10^5^ cells/mL in RPMI medium supplemented with 10% FBS containing 2 μg/mL of Con A in the absence or presence of mDC or tDCs (1:10 ratio) for 72 h. Quantitative evaluation of the exponential cell expansion was estimated by the carboxyfluorescein succinimidyl ester–CFSE assay (Invitrogen/Molecular Probes). CFSE staining was performed according to methodology previously described (27). Before acquisition, cells were centrifuged and the pellet was washed twice with cold PBS and labeled with APC anti-mouse CD3 (Biolegend, San Diego, CA) diluted 1:100 for 15 min. Acquisition was performed using a BD LSRFortessa SORP cytometer and data were analyzed using FlowJo software (Tree Star, Ashland, OR). A total of 100,000 events were acquired.

### T Regulatory Cells Quantification

For quantification of T regulatory cells, splenocytes cultured with Con A (2 μg/mL) in the absence or presence of mDCs or tDCs were stained to CD4^+^ CD25^+^ Foxp3^+^ Treg cells using the Mouse Regulatory T Cell Staining Kit (eBioscience), according to the manufacturer's recommendations. Briefly, 10^6^ splenocytes were stained with anti-CD4-FITC and anti-CD25-APC, followed by permeabilization with cold Fix/Perm Buffer and blocking with anti-mouse CD16/32. Then, anti-Foxp3-PE (eBioscience) was added for intracellular Foxp3-staining. Labeled cells were analyzed using a LSRFortessa cytometer and FlowJo software.

### Immunotherapy With tDCs

Groups of 10 chronic chagasic mice (3 months post-infection) were treated with four monthly intraperitoneal injections of 5 × 10^5^ tDCs, or equal volume of saline (100 μL). The DC were generated independently for each intraperitoneal injection. Two weeks after the last injection, mice were euthanized under anesthesia with 5% ketamine (Vetanarcol® Konig, Avellaneda, Argentina) and 2% xylazine (Sedomin® Konig). Hearts were collected and divided into two halves, being one frozen at −80°C and the other fixed in 10% buffered formalin, for PCR and histopathology, respectively. Spleens were frozen in Tissue-Tek (Sakaru, Alphen a den Rijn, The Netherlands), for immunofluorescence analysis.

### Histology and Morphometric Analyses

Heart sections were analyzed by light microscopy after paraffin embedding, followed by standard hematoxylin and eosin (H&E), and Sirius red staining methods for evaluation of inflammation and fibrosis, respectively, by optical microscopy. Images were digitized using a color digital video camera (CoolSnap, Montreal, Canada) adapted to a Bx41 microscope (Olympus, Tokyo, Japan). Morphometric analyses were performed using the software Image Pro Plus v.7.0 (Media Cybernetics, San Diego, CA). The inflammatory cells were counted in 10 randomly captured fields (x400 view). The percentage of fibrosis was determined using Sirius red-stained heart sections and Image-Pro Plus v.7.0 to integrate the areas, 10 fields per animal were randomly captured using x200 view. Analyses were performed in a blinded fashion.

### Real-Time Reverse Transcription Polymerase Chain Reaction (RT-qPCR)

RNA was extracted of the heart samples using TRIzol (Invitrogen-Molecular Probes, Eugene, OR). The cDNA was synthetized using the High Capacity cDNA Reverse Transcription KIT (Applied Biosystems, Foster City, CA). The qPCR was prepared with TaqMan® Universal PCR Master Mix (Applied Biosystems). qRT-PCR assays were performed to detect the expression levels of *Il12* (Mm_00434165_m1), *Col1a2* (Mm_00483888_m1), *Lgals3* (Mm_00802901_m1), *Il10* (Mm_00439616_m1), *Ifng* (Mm_00801778_m1), and *Foxp3* (Mm_00475162_m1). All reactions were run in triplicate on an ABI 7500 Real Time PCR System (Applied Biosystems) under standard thermal cycling conditions. A non-template control (NTC) and non-reverse transcription controls (No-RT) were also included. The samples were normalized with *Hprt* (Mm_00484683_m1). The threshold cycle (2-ΔΔCt) method of comparative PCR was used to analyze the data (28).

### Immunofluorescence Analysis

Sections of formalin-fixed paraffin-embedded hearts and frozen spleens were used for detection of CD3 and Foxp3 expression by immunofluorescence. First, paraffin-embedded sections were deparaffinized and submitted to a heat-induced antigen retrieval step by incubation in citrate buffer (pH 6.0). Then, sections were incubated overnight with the following primary antibodies: anti-CD3 (1:400; BD Biosciences) and anti-Foxp3 (1:400; Dako, Glostrup, Denmark). On the following day, secondary antibodies anti-goat IgG Alexa Fluor 488 (1:600; Molecular Probes) or anti-rabbit IgG Alexa Fluor 568 conjugated (1:100; Molecular Probes), diluted in 1% BSA in PBS, were added. Nuclei were stained with 4,6-diamidino-2-phenylindole (DAPI; VectaShield Hard Set mounting medium with DAPI H-1500; Vector Laboratories, Burlingame, CA). Images were analyzed using a confocal laser scanning microscope A1R (Nikon, Tokyo, Japan) and Image-Pro Plus version 7.01 (Media Cybernetics, Rockville, MD). Quantifications of CD3^+^/Foxp3^+^ cells percental area were performed in 10 fields randomly captured under x400 magnification, using Image-Pro Plus v.7.0.

### Statistical Analyses

The normality of the data was determined by the Shapiro-Wilk normality test. In order to analyze differences among groups, the one-way analysis of variance test followed by the Newman Keuls test was used for parametric data and the Kruskal-Wallis test followed by Dunn's post-test was used for nonparametric data. To compare the means of the two groups, Mann-Whitney's *U* test for nonparametric data and Student's *t* test for parametric data were used. All analyses were performed using Prism version 5.01 (GraphPad Software, San Diego, CA). All differences were considered significant at values of *p* < 0.05.

## Results

### Characterization of Tolerogenic Dendritic Cells (tDCs)

**Figure 1:**
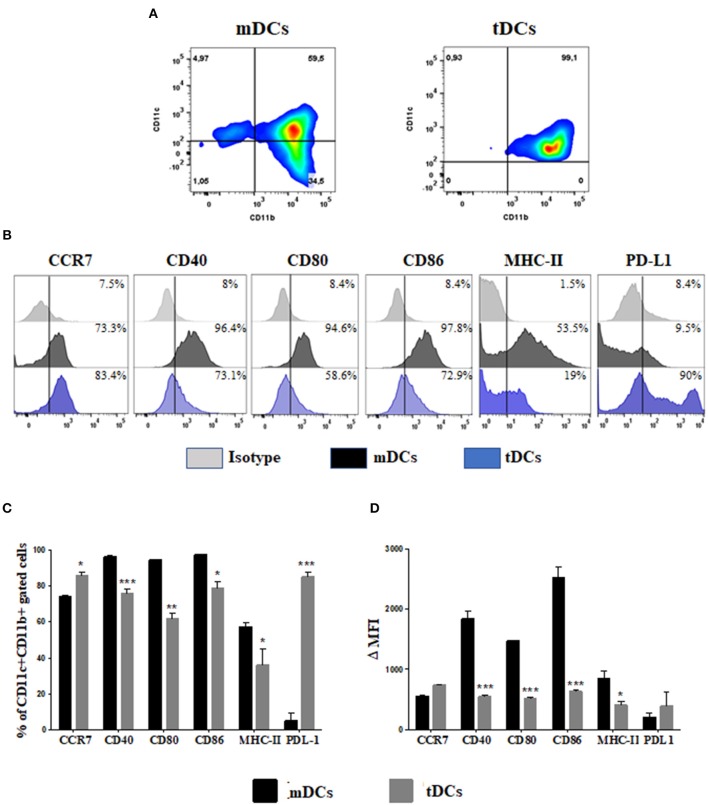
Immunophenotyping of differentiated DCs from bone marrow cells in the presence of dexamethasone. **(A)** Dot plot representing the percentage of CD11c^+^ CD11b^+^ cells. **(B)** Histograms showing the expression of CCR7, CD40, CD80, CD86, MHC-II, and PD-L1 in mDCs (black) and tDCs cells (blue), within the population of CD11c^+^ CD11b^+^ cells. **(C)** The percentage of costimulatory molecules gated on CD11c^+^ CD11b^+^ cells. **(D)** The Δ mean fluorescence intensity (MFI) of costimulatory molecules gated on CD11c^+^ CD11b^+^ cells. MHC, major histocompatibility complex; PD-L, programmed death ligand. Values represent the mean of nine determinations ± SEM of three experiments performed. ***P < 0.001; compared to mDC cells. **P < 0.01; compared to mDC cells. *P < 0.05; compared to mDC cells.

**Figure 2:**
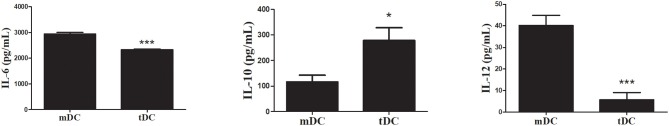
TDCs exhibit an anti-inflammatory cytokine secretion profile. Concentrations of IL-6 **(A)**, IL-10 **(B)**, and IL-12p70 **(C)** were determined in mDCs or tDCs supernatants by ELISA. Values represent the mean of nine determinations ± SEM of three experiments performed. ***P < 0.001; compared to mDC cells. *P < 0.05; compared to mDC cells.

Initially, we determined the phenotype of the tDCs generated in the presence of dexamethasone for induction of tolerogenic profile (Figure 1). The analysis by flow cytometry of CD11b^+^ CD11c^+^ population showed a lower expression of CD40, CD80, CD86, and MHC-II in tDCs (Figures 1A–C). This was confirmed by significantly loss in the mean intensity fluorescent (ΔMFI) of these markers in tDCs (Figure 1D). In contrast an increase expression of CCR7 and PD-L1, not accompanied by a significant increase in ΔMFI, was observed in cultures of tDCs compared to mDCs (Figures 1B–D). Additionally, the tDCs produced lower levels of IL-6 and IL-12, and higher levels of IL-10, compared to mDCs (Figures 2A–C). These results indicate a regulatory phenotype of tDCs generated from bone marrow cells by treatment with dexametosone *in vitro*.

### Modulation of Cell Proliferation in vitro by tDCs

**Figure 3:**
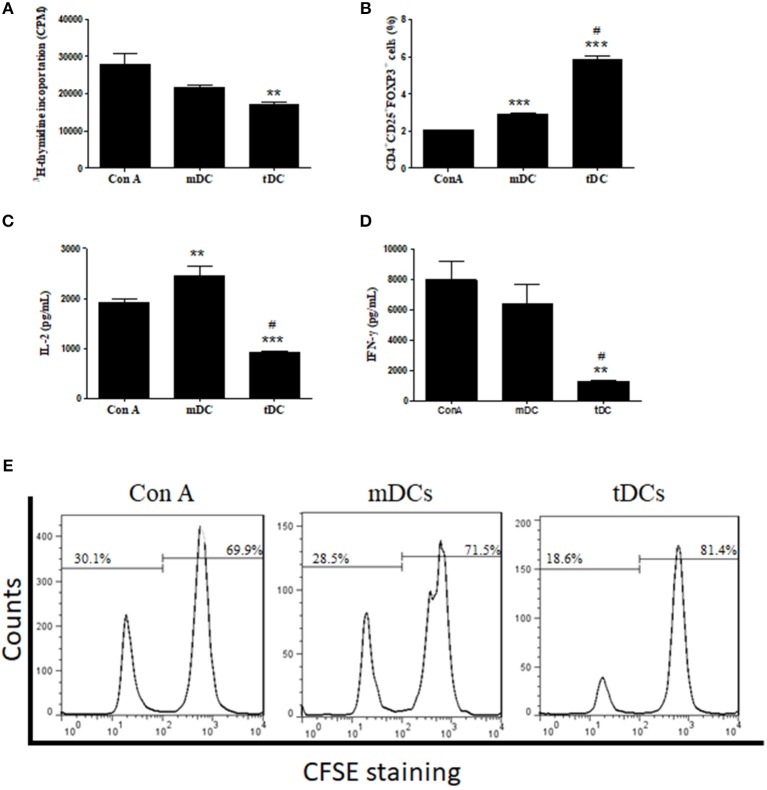
Inhibition of lymphocyte proliferation and stimulation of FoxP3^+^ Treg cells. **(A)** Effect of mDCs or tDCs on ConA-induced lymphoproliferation for 48 h. **(B)** Effect of mDCs or tDCs in induction of T regulatory (Treg) cells. **(C,D)** Concentrations of IL-2 and IFN-γ determined by ELISA. **(E)** Effect of mDCs or tDCs on Con-A induced lymphoproliferation evaluated by CFSE staining. Values represent the mean of 9 or 12 determinations ± SEM of three experiments performed. ***P < 0.001; compared to Con A group. **P < 0.01; compared to Con A group. ^#^*P* < 0.001; compared to mDC group.

To demonstrate a modulatory effect of tDCs in lymphocyte activation, a lymphoproliferation assay was performed using splenocytes from *T. cruzi*-infected mice stimulated with concanavalin A. As shown in Figure 3A, the tDCs caused a significant inhibition of lymphoproliferation, while the addition of mDCs did not cause a significant inhibition. Similarly, by using CFSE staining, a marker of cell proliferation, we found that tDCs, but not mDCs, are able to inhibit Con A-induced lymphoproliferation (Figure 3E). Interestingly, addition of tDCs also increased the population of FoxP3^+^ Treg cells, compared to the control mDCs (Figure 3B). Concomitantly, tDCs, but not mDCs, caused a significant reduction in IL-2 and IFN-γ cytokine production (Figures 3C,D).

### Immunotherapy With Tolerogenic Dendritic Cells Prevents Progression of CCC

**Figure 4:**
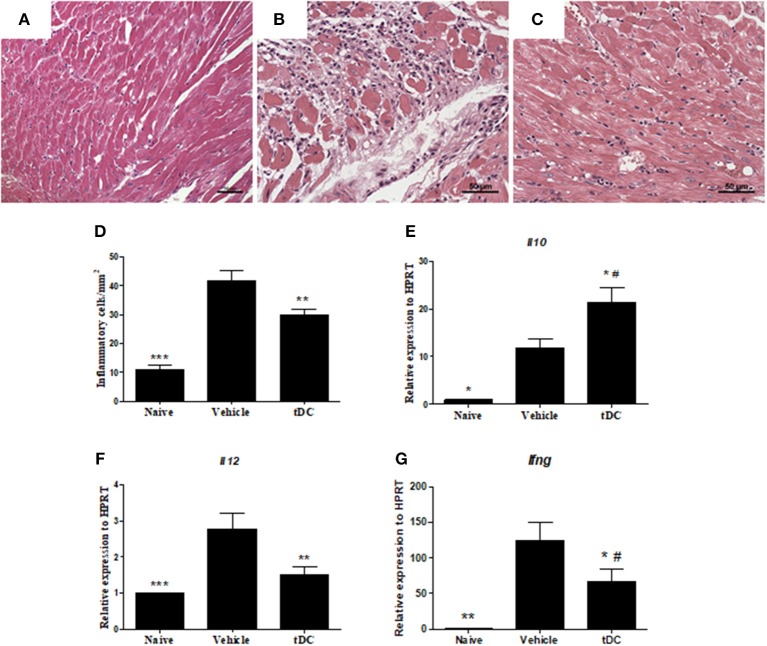
Reduction of inflammation and quantification of the gene expression of pro and anti-inflammatory cytokines in the hearts of tDCs-treated mice. **(A–C)** Micrographs of hematoxylin and eosin-stained heart sections of naïve **(A)**, vehicle-treated **(B)** and tolerogenic dendritic cells-treated **(C)** groups. **(D)** The number of inflammatory cells was quantified by morphometrical analysis. Gene expression of anti-inflammatory IL-10 **(E)** and proinflammatory IL-12 **(F)** and IFNγ **(G)** cytokines were assessed by RT-qPCR using cDNA samples prepared from mRNA extracted from mouse hearts from the experimental groups. Values represent the mean of at least 16 determinations ± SEM of two experiments performed with 8–10 mice/group ***P < 0.001; compared to vehicle-treated mice. **P < 0.01; compared to vehicle-treated mice. *P < 0.05; compared to vehicle-treated mice ^#^*P* < 0.05; compared to naïve mice.

The therapeutic effects of tDCs was then evaluated in a mouse model of CCC. Heart sections were examined after stained with hematoxylin and eosin and Sirius red for quantification of inflammation and fibrosis, respectively. A multifocal inflammation, mainly composed by mononuclear cells, was found in the hearts of saline-treated *T. cruzi*-infected mice compared to naïve mice (Figures 4A,B). The administration of tDCs led to a significantly reduction in the number of inflammatory cells compared to saline-treated mice (Figures 4C,D).

Mice chronically infected with *T. cruzi* had an increased production of inflammatory mediators in the heart, compared to naïve mice (Figure 4). In the hearts of tDCs treated mice, however, the analysis of gene expression showed a reduction of the pro-inflammatory cytokines IL-12 and IFN-γ, while IL-10 expression was increased, when compared to vehicle-treated mice (Figures 4E–G).

**Figure 5:**
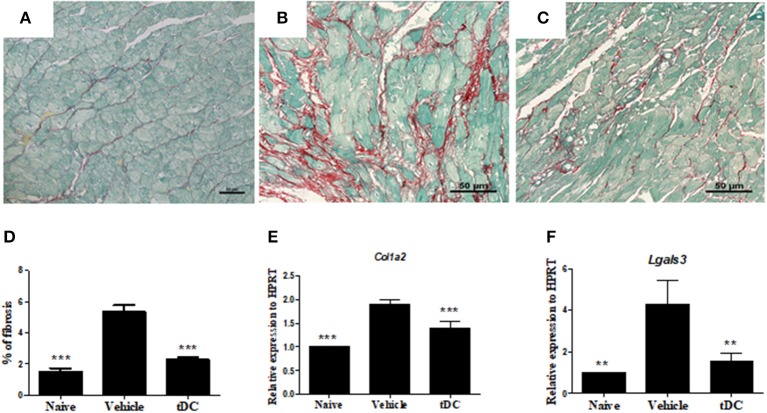
Reduction of fibrosis and fibrosis-associated factors in the hearts of mice treated with tDCs. **(A–C)** Heart sections stained with Sirius-red from naïve **(A)**, vehicle-treated **(B)** and tolerogenic dendritic cells-treated **(C)** groups. **(D)** The fibrosis area is represented by the percentage of collagen deposition in the heart sections. Gene expression of collagen 1a2 **(E)** and galectin 3 **(F)** was assessed by RT-qPCR using cDNA samples prepared from mRNA extracted from hearts from experimental groups. Values represent the mean of at least 16 determinations ± SEM of two experiments performed with 8–10 mice/group. ***P < 0.001; compared to vehicle-treated mice. **P < 0.01; compared to vehicle-treated mice.

Similarly, infected mice presented a higher percentage of fibrosis in the heart when compared to naïve controls (Figures 5A,B). Treatment with tolerogenic dendritic cells also promoted a reduction in the percentage of fibrosis when compared with saline-treated mice (Figures 5C,D). This was correlated with a reduction on gene expression of collagen 1a2 and galectin 3, two factors related to fibrogenesis, as shown by RT-qPCR (Figures 5E,F).

### Administration of Tolerogenic Dendritic Cells Increases the Percentage of Treg Cells in the Hearts and Spleens of Chagasic Mice

**Figure 6:**
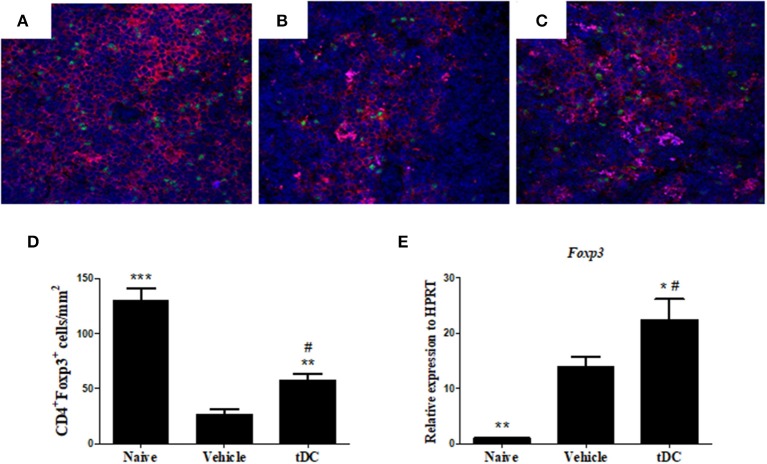
Treatment with tDCs increases the expression of Tregs for spleen and heart. **(A–C)** Frozen spleen sections obtained from mice from each experimental group were stained with antibodies specific for CD4 (red), FoxP3 (green), and DAPI for nuclei staining (blue) in the spleen tissue of naïve **(A)**, vehicle-treated **(B)** and tDCs-treated **(C)** mice. **(D)** Quantification of spleen sections stained for CD4^+^ and FoxP3^+^. DAPI, 4′, 6-Diamidino-2-phenylindole. The gene expression of FoxP3 **(E)** was evaluated by RT-qPCR using cDNA samples prepared from mRNA extracted from hearts from experimental groups. Values represent the mean of at least 16 determinations ± SEM of two experiments performed with 8–10 mice/group. ***P < 0.001; compared to vehicle-treated mice. **P < 0.01; compared to vehicle-treated mice. *P < 0.05; compared to vehicle-treated mice ^#^*P* < 0.001; compared to naïve mice.

One of the effector mechanisms by which tolerogenic dendritic cells act is the induction of Treg cells (24). We next evaluated whether the administration of tolerogenic dendritic cells induces the increase of Treg cells in *T. cruzi*-infected mice. FoxP3 expression was investigated in CD4^+^ cells in spleen sections, by immunofluorescence. The percentage of CD4^+^ FoxP3^+^ Treg cells increased markedly in the spleens of mice treated with tolerogenic dendritic cells, compared with the group saline-treated mice (Figures 6A–D). Concomitantly, an increase in the level of FoxP3 mRNA was observed in the hearts of tolerogenic dendritic cells-treated mice, compared with that of the group treated with saline (Figure 6E).

## Discussion

CCC is characterized by persistent inflammation leading to extensive cardiac, with progressive damage to the myocardium and heart dysfunction (29, 30). In the present study, the use of tolerogenic dendritic cells as a therapeutic tool for CCC was examined using mouse model. Our results show that administration of tolerogenic dendritic cells promoted reduction of heart inflammation and fibrosis, two important features of Chagas heart disease.

We generated tolerogenic dendritic cells in the presence of GM-CSF by treating *in vitro* with dexamethasone, followed by LPS-induced maturation. The multifunctional cytokine GM-CSF modulates the growth and function of most leukocytes and plays a key role in mediating the differentiation and growth of DCs from mononuclear cells (31). The use of dexamethasone as an agent for the induction of tolerogenic profile in DCs has been previously shown in both mouse bone marrow cells and from human monocytes (32–34). Tolerogenic dendritic cells derived by the use of pharmacological agents, such as dexamethasone alone or in combination, and subsequently induced maturation with LPS has previously been shown to acquire stable semi-mature and tolerogenic phenotype (35–38). Furthermore, the use of LPS to generate tolerogenic dendritic cells has proved to be important to favor the regulatory and migratory capacity of these cells (34). In our study, this protocol generated tDCs with low expression of CD40, CD80, CD86, and MHC-II costimulatory molecules, and low production of the proinflammatory cytokines IL-6 and IL-12, while presenting increased production of IL-10. Altogether, these are characteristics compatible with an immunosuppressive profile (39, 40).

The reduction of IL-12 is highly related to the increase of IL-10 production (36). This is a characteristic profile of tolerogenic dendritic cells generated from dexamethasone, which may also have the ability to induce Treg cells (35, 36). The immune tolerance naturally can be achieved by different mechanisms, including T cell anergy, the elimination of self-reactive T cells or the induction of Treg cells (36, 40). Our results suggest that the tolerance induction by tDCs tested here occurs by induction of Treg cells belonging to the subset CD4^+^CD25^+^ expressing FoxP3^+^. The expression of FoxP3^+^ and differentiation of Treg cells occurs only in the presence of DC or IL-10, characterizing positive feedback regulations (24, 36, 41–43).

Importantly, the tDCs significantly reduced the inflammatory infiltrate in the hearts of mice with CCC. This effect is probably caused by the immunosuppressive profile of tolerogenic dendritic cells, which have low immunostimulatory capacity, but with potential of inducing Treg cells via IL-10 secretion. As shown by recent studies, tolerogenic dendritic cells producing IL-10 induce tolerance via activation of Treg cells in murine models of autoimmune disease (22, 34, 35). Interestingly, in the absence of DCs, IL-10 is a cytokine which alone can induce FoxP3 expression and Treg differentiation (42). The capacity to produce IL-10 could therefore be one of the mechanisms by which tolerogenic DCs inhibit the progression of CCC, through its systemic effect, in addition to inducing the differentiation of FoxP3^+^Treg cells (25, 36, 41, 42). Moreover, we found a significant reduction in IL-12 and IFNγ gene expression in the hearts of tDCs-treated mice. The reduction of IFNγ expression is also related to the reduction of the inflammatory infiltrate, since high levels of this inflammatory cytokine induce the activation of macrophages through the high production of IL-12 and toxic intermediates, such as nitric oxide (NO) and reactive oxygen intermediates (ROI), consequently leading to the activation of a polarized Th1 response (44, 45). Therefore, tDCs may also affect the maturation of DCs and macrophages, since the secretion of IL-10 interferes with the up-regulation of costimulatory molecules and inhibits the IL-12 and IFNγ production, thus limiting the ability to promote Th1 responses (44–48).

Another important action of tolerogenic dendritic cells in the CCC was the reduction of fibrosis, which was associated with a reduction in the expression levels of col1a2 and galectin 3 genes after treatment with tDCs. The role of Galectin 3 in the process of cardiac remodeling has been shown in experimental models of cardiomyopathy hypertrophy and myocardial infarction (49, 50). In CCC, our previous reports have shown that Gal-3 expression correlates with the degree of inflammation and fibrosis in the heart (12, 13, 51). Moreover, the pharmacological blockage of this protein caused reduction of cardiac inflammation and fibrosis in infected mice (52). Thus, the reduction of fibrosis and collagen production observed after therapy with tDCs may be due to the downmodulation on galectin 3 expression.

Ours results show that tolerogenic dendritic cells have therapeutic effect on CCC, inhibiting disease progression. Positive effects were observed in the absence of specific antigen-pulsed tolerogenic dendritic cells. Immunotherapy with tolerogenic dendritic cells increased the levels of FoxP3^+^ Treg cells in the heart and spleen, implying that Treg cells promote immune modulation leading to inhibition of CCC progression.

In conclusion, this study is the first to show that treatment with bone marrow-derived tolerogenic dendritic cells effectively improves myocardial inflammation and fibrosis in murine model of chronic chagasic cardiomyopathy. This suggests the use of tolerogenic dendritic cells as a promising therapeutic strategy for treating CCC.

## Data Availability Statement

The raw data supporting the conclusions of this article will be made available by the authors, without undue reservation, to any qualified researcher.

## Ethics Statement

The animal study was reviewed and approved by Animal Ethics Commission of Gonndations of Ethical Issues Guideli (Approved number: 017/2017).

## Author Contributions

ES, LA-F, CM, JC, JV, and CN performed the experiments. ES, LA-F, CM, LP-C, and MS analyzed the data. ES, LA-F, LP-C, and MS conceived the study and wrote the manuscript.

### Conflict of Interest

The authors declare that the research was conducted in the absence of any commercial or financial relationships that could be construed as a potential conflict of interest.
